# Phase 2 study of AV-GBM-1 (a tumor-initiating cell targeted dendritic cell vaccine) in newly diagnosed Glioblastoma patients: safety and efficacy assessment

**DOI:** 10.1186/s13046-022-02552-6

**Published:** 2022-12-14

**Authors:** Daniela A. Bota, Thomas H. Taylor, David E. Piccioni, Christopher M. Duma, Renato V. LaRocca, Santosh Kesari, Jose A. Carrillo, Mehrdad Abedi, Robert D. Aiken, Frank P. K. Hsu, Xiao-Tang Kong, Candace Hsieh, Peter G. Bota, Gabriel I. Nistor, Hans S. Keirstead, Robert O. Dillman

**Affiliations:** 1 University of California Irvine Department of Neurology and Chao Family Comprehensive Cancer Center, Orange, USA; 2 Department of Epidemiology & Biostatistics and Chao Family Comprehensive Cancer Center, University of California, Irvine, Irvine, United States; 3 University of California San Diego, La Jolla, USA; 4 Hoag Hospital and Hoag Neuroscience Institute, Newport, USA; 5 Norton Cancer Institute, Louisville, USA; 6 John Wayne Cancer Institute, and Pacific Neuroscience Institute, Santa Monica, USA; 7 University of California Davis, Davis, USA; 8 Rutgers Cancer Center, Brunswick, USA; 9 AIVITA Biomedical, Inc., Irvine, USA; 10 California University of Science and Medicine, Colton, USA

**Keywords:** Glioblastoma, Dendritic cell vaccine, Autologous tumor antigens, Survival

## Abstract

**Background:**

Vaccine immunotherapy may improve survival in Glioblastoma (GBM). A multicenter phase II trial was designed to determine: (1) the success rate of manufacturing the Aivita GBM vaccine (AV-GBM-1), (2) Adverse Events (AE) associated with AV-GBM-1 administration, and (3) survival.

**Methods:**

Fresh suspected glioblastoma tissue was collected during surgery, and patients with pathology-confirmed GBM enrolled before starting concurrent Radiation Therapy and Temozolomide (RT/TMZ) with Intent to Treat (ITT) after recovery from RT/TMZ. AV-GBM-1 was made by incubating autologous dendritic cells with a lysate of irradiated autologous Tumor-Initiating Cells (TICs). Eligible patients were adults (18 to 70 years old) with a Karnofsky Performance Score (KPS) of 70 or greater, a successful TIC culture, and sufficient monocytes collected. A cryopreserved AV-GBM-1 dose was thawed and admixed with 500 μg of Granulocyte-Macrophage Colony-Stimulating Factor (GM-CSF) before every subcutaneous (s.c.) administration.

**Results:**

Success rates were 97% for both TIC production and monocyte collection. AV-GBM-1 was manufactured for 63/63 patients; 60 enrolled per ITT; 57 started AV-GBM-1. The most common AEs attributed to AV-GBM-1 were local injection site reactions (16%) and flu-like symptoms (10%). Treatment-emergent AEs included seizures (33%), headache (37%), and focal neurologic symptoms (28%). One patient discontinued AV-GBM-1 because of seizures. Median Progression-Free Survival (mPFS) and median Overall Survival (mOS) from ITT enrollment were 10.4 and 16.0 months, respectively. 2-year Overall Survival (OS) is 27%.

**Conclusions:**

AV-GBM-1 was reliably manufactured. Treatment was well-tolerated, but there were numerous treatment-emergent central nervous system AEs. mPFS was longer than historical benchmarks, though no mOS improvement was noted.

**Trial registration:**

NCT, NCT03400917, Registered 10 January 2018,

**Supplementary Information:**

The online version contains supplementary material available at 10.1186/s13046-022-02552-6.

## Background

GBM is a highly lethal brain malignancy most frequently occurring in older adults [1, 2]. Standard-of-Care (SOC) therapy for newly-diagnosed Glioblastoma (nGBM) patients includes maximum safe resection and concurrent RT/TMZ followed by maintenance Temozolomide (TMZ). In four large randomized clinical trials conducted during the past 20 years, median mOS respectively were 14.6, 16.1, 16.7, and 16.6 months; 24-month OS was 26, 29, 27, and 28%, and mPFS was 6.9, 7.3, 6.2, and 5.5 months [3–6].

Adjunctive vaccine immunotherapy may improve survival by enhancing a patient’s anti-GBM immune response and, recently, interest has emerged in using autologous Dendritic Cells (DCs) to present Anti-Tumor Antigens (ATAs) in such vaccines, as tumor heterogeneity precludes the use of shared antigens [7]. Dendritic Cell Anti-Tumor Antigen (DC-ATA) vaccines utilizing autologous antigens from short-term self-renewing cell cultures have been safely administered in phase I-II trials in patients with melanoma [8–13], hepatocellular cancer [14], and renal cell cancer [15], which also suggested long-term survival and delayed complete regressions of metastatic disease [9, 11, 13, 15–17].

The DC-ATA approach requires fresh tumor tissue to establish cell cultures of self-renewing tumor cells [18]. These cultures contain TICs, which are believed to play some part in chemo-resistance [19–21]. nGBM is an excellent target for this approach because surgical resection is part of SOC. A previous trial in patients with recurrent GBM demonstrated that DC pulsed with ATA could be safely administered in combination with TMZ [22]. A multicenter, single-arm phase II trial was designed to determine: (1) the success rate of manufacturing the DC-ATA vaccine AV-GBM-1, (2) AEs associated with AV-GBM-1 administration in combination with SOC, and (3) OS and PFS.

## Methods

Key eligibility criteria at the time of ITT enrollment were pathological diagnosis of GBM (Grade IV WHO astrocytoma) [23], KPS of 70 or higher, age 18 to 70, and adequate organ function. In addition, eligibility required a successful short-term cell culture of autologous TICs and collection of sufficient numbers of monocytes by leukapheresis. Key exclusion criteria were a previous diagnosis of glioma, underlying disease process (e.g., invasive cancer, advanced heart disease) considered to be life-threatening within the next five years, active infection, active treatment with immunosuppressive agents other than 4 mg dexamethasone (or equivalent), pregnancy or nursing (for full inclusion and exclusion Criteria, see Supplementary Data 2). There were no exclusion criteria based on radiographic findings post-concurrent RT/TMZ, or by whether patient was felt to already have progressive disease. (because of inability to clearly distinguish Progressive Disease (PD) from Pseudo-Progressive Disease (PsPD). Overall survival from the date of intent-to-treat enrollment was the primary endpoint and was the basis for determining the number of patients to be enrolled. Secondary efficacy endpoints were PFS from date of ITT enrollment, and OS and PFS from date of first injection. Safety endpoints were enumerations of AE and SAE from the start of treatment. As a result, *N* = 60 for ITT assessment, *N* = 57 for patients who initiated treatment.

Detailed descriptions of DC-ATA manufacturing were published previously [8, 9, 11, 13], with the current trial using of serum-free media to favor the establishment of TICs and tumor cell lysate as the antigen source for incubation (see Supplementary Data 1 for the production method and Supplementary Data 3 for an explanation of the reasoning behind how TICs are isolated). Each patient’s DC-ATA batch was divided into ten equal aliquots in single-dose vials in a storage container in the vapor phase of liquid nitrogen (−190 °C to −150 °C). Each dose of AV-GBM-1 was shipped to the treatment site in a liquid nitrogen dewar, admixed with 500 μg GM-CSF (Leukine®, Partner Therapeutics), and subsequently injected s.c. within five hours of thawing.

The patients received three weekly s.c. injections of AV-GBM-1 (week 1, 2, and 3) after the completion of concurrent RT/TMZ, and standard TMZ-based maintenance therapy could commence one week after the third dose. An additional five AV-GBM-1 injections were administered at weeks 8, 12, 16, 20, and 24. If/when patients were felt to have progressive disease while receiving AV-GBM-1, they were permitted to continue the vaccine and add additional standard therapies at the discretion of the managing physician. Baseline history and physical examination were obtained during eligibility screening and at the time of ITT enrollment prior to starting RT/TMZ. Neurological assessments were performed at each visit. Complete blood counts, chemistries, and MRI scans were ordered per SOC. AEs were identified and classified per Common Terminology Criteria for Adverse Events (CTCAE v 4.03). Severe Adverse Events (SAEs) were collected separately. A final safety assessment was made 28 days after the last injection of the vaccine; subsequent follow-up was at 3-month intervals from the ITT date.

The individual investigators defined PD using the iRANO criteria [24]. There was no central review of MRI scans.

The *primary objectives* were to determine: 1. the success rate for manufacturing AV-GBM-1, 2) the frequencies and severity of Treatment-Emergent Adverse Events (TEAE), and 3) OS calculated from the date of ITT enrollment pre-RT/TMZ. *Secondary objectives* included PFS from the date of ITT enrollment, and both OS and PFS calculated from the date of first injection (which was after RT/TMZ). *Tertiary (exploratory objectives)* included analysis of OS and PFS in subpopulations defined by age 60 years or greater vs. 59 or less, KPS of 90 or 100 vs. 70 or 80, O-6-methylguanine-DNA methyltransferase *(MGMT)* promoter methylated vs unmethylated, and Isocitrate Dehydrogenase (*IDH1/2)*-mutated vs. *IDH1/2* wild-type.

### Study design and statistical analysis

This was a single-arm phase 2 clinical trial. The primary research hypothesis was that the addition of vaccine to SOC would yield an OS of 75% at 14.6 months, compared to 50% in the benchmark study [5]. Our accrual goal of 55 evaluable has over a 90% chance of yielding an 80% confidence interval whose lower bound exceeds the upper bound of the 95% confidence limit we estimated for the benchmark OS result, given the OS is 75%. We compare our PFS results to the benchmark mPFS of 6.9 months [5]. The Kaplan-Meier estimator was used to generate PFS and OS curves for both ITT and Target Product Profile (TPP) populations [25]; 95% confidence intervals (CI) were estimated for the PFS and OS determinations. Unadjusted Mantle-Cox log-rank tests were used to compare survival curves in exploratory subset analyses. Chi-square or Fishers Exact Test were used to compare proportions.

## Results

### Conduct of study

Patients were enrolled from five sites in California and one each in Kentucky and New Jersey. Tumors were collected between August 2018 and January 2020, with ITT enrollment occurring between September 2018 and February 2020. TPP vaccine injections took place from November 2018 to October 2020.

**Fig. 1:**
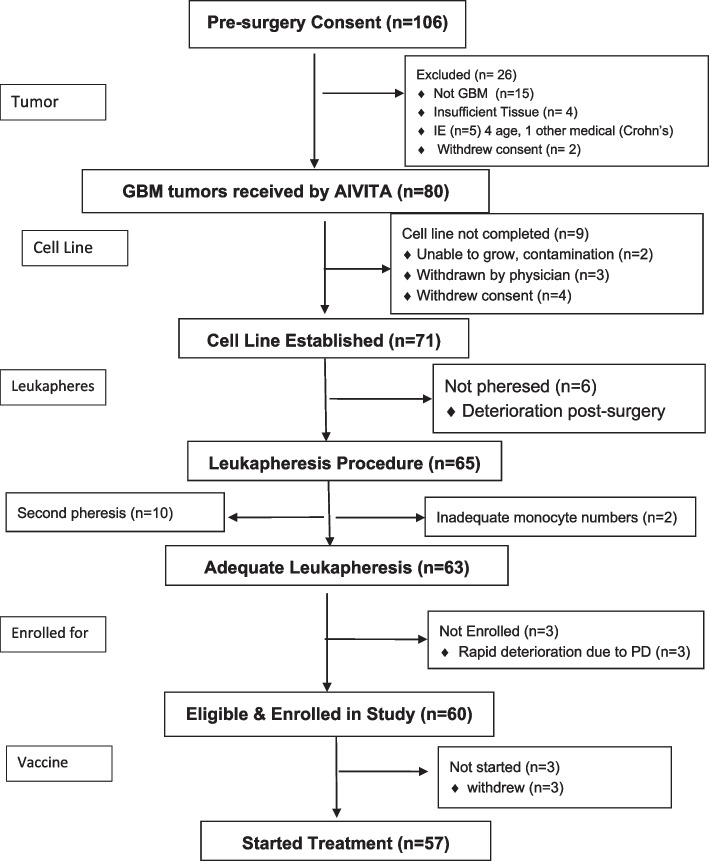
Flow diagram of AV-GBM-1 nGBM trial (Reformatted)

As of 31 Dec 2021, 39 of the 60 ITT patients had died, and 21 remained alive at last contact. Of the 21 alive, 18 had been at risk of death for a minimum of 15.2 months from ITT enrollment. Of the 57 who received at least one dose of AV-GBM-1, 38 were deceased and 19 were alive at last contact. The flow from screening to cell collection to enrollment and treatment is shown in Fig. 1. Because not all the tumor donors were eligible to proceed with the AV-GBM-1 treatment, tumor collection was not discontinued until it was certain that at least 55 patients would be enrolled per ITT. As a result, products were actually made for 60/60 of those who completed ITT enrollment, including 57 who received a vaccine injection. The mean and median time from surgery to ITT enrollment date was 1.0 months (range 0.2 to 3.0), and from enrollment date to first treatment date was 2.2 months (range 1.4 to 3.4 months).

### Feasibility

As shown in Fig. 1, the success rate for establishing a tumor cell line for each patient was 97%, identical to success rate for the collection of sufficient monocytes, albeit with 10 patients requiring a second pheresis procedure. AV-GBM-1 product was manufactured for 60/60 (100%) of ITT-enrollees (for more details, see Supplementary Data 1). The numbers of cells for intermediate and final products are shown in Table 1.

**Table 1:** Characteristics of AV-GBM-1 products manufactured for patients (*n* = 60)

Variable	Mean	SEM	Median	Lower Limit	Upper Limit
Total irradiated tumor cells x 10^6^	14.0	1.2	11.0	0.78	63.4
Number of monocytes frozen x 10^9^	1.7	0.15	1.5	0.075	5.2
Number of DC to incubate with ATA x 10^6^	750	108	560	38	5720
Total DC-ATA per dose x 10^6^	7.9	0.68	7.4	0.26	27.0
% DC-ATA viable at cryopreservation	80.8	1.7	84.0	48.9	100

### Patient characteristics

Table 2 shows the characteristics of patients and their tumors. Methylation of the *MGMT* gene promoter was present in 21 (35%) patients, and *IDH1/2* mutations were present in 7 patients (11.7%). Local sites reported gross total resection of GBM for 55 patients (92%).

**Table 2:** Patient Characteristics

Variable	*N* = 60 (100%)
Age	
Median	59 years
Range	29 to 70 years
Gender	
Male	42 (70%)
Female	18 (30%
Race/Ethnicity	
Asian	1 (1.7%)
Black	2 (3.3%)
Hispanic	10 (17%)
Other	3 (5.0%)
White	43 (72%)
KPS at baseline	
70	14 (23%)
80	17 (28%)
90	25 (42%)
100	4 (7%)
MGMT promoter status	
Methylated	20 (33%)
Unmethylated	39 (65%)
Equivocal	1 (2%)
IDH1/2 genes	
Mutated	7 (12%)
Not Mutated	53 (88%)
Anatomic Location of Tumor	
Frontal	Left 11 (18%), Right 5 (8.3%)
Parietal	Left 6 (10%), Right 8 (13%)
Temporal	Left 7 (12%), Right 13 (22%)
Occipital	Left 2 (3.3%), Right 1 (1.7%)
Fronto-Temporal	Left 0 (0%), Right 1 (1.7%)
Parieto-Occipital	Left 1 (1.7%) Right 0 (0%)
Temporo-Occipital	Left 0 (0%) Right 1 (1.7%)
Temporo-parieto-occipital	Left 1 (1.7%), Right 0 (0%)
Temporal and Amygdala	Left 0 (0%), Right 1 (1.7%)
Insula	Left 1 (1.7%), Right 0 (0%)
Corpus callosum	Left 1 (1.7%), Right 0 (0%)

### Vaccine dosing and concomitant therapies (Table 3)

Sixty patients were enrolled per ITT, but three withdrew before starting the treatment with AV-GBM-1. During the first three weeks of vaccination, no concurrent anti-cancer therapy was given. After receiving three weekly AV-GBM-1 injections, concurrent, adjuvant TMZ-based therapy was administered to 49 patients, while 8 patients stopped treatment before starting adjuvant TMZ. Our protocol also allowed the use of the standard treatments for nGBM, including Tumor-Treating Fields (TTF) [26] and bevacizumab (only to control radiation-induced edema) [27]. Concurrent therapy in our cohort included: TMZ alone (*n* = 28), TMZ + bevacizumab (*n* = 11), TMZ + TTF (*n* = 7), and TMZ + bevacizumab + TTF (*n* = 3). Forty patients took corticosteroids concurrently with AV-GBM-1.

**Table 3:** Concurrent therapy and prognostic factors

	*MGMT* (methylated, not methylated)	*IDH* (mutated, not mutated)	Age (years) (59 or less, > 60)	KPS (90 or 100, 70 or 80)
No vaccine (*n* = 3)	1 vs 2	0 vs 3	1 vs 2	2 vs 1
2 or 3 vaccinations, no concurrent therapy (*n* = 8)	3 vs 5	1 vs 7	4 vs 4	1 vs 7
Concurrent TMZ alone (*n* = 29)	8 vs 21	4 vs 25	18 vs 11	17 vs 12
Concurrent TMZ + bevacizumab (*n* = 11)	3 vs 8	2 vs 9	7 vs 4	6 vs 5
Concurrent TMZ + TTF (*n* = 6)	3 vs 3	0 vs 6	2 vs 4	3 vs 3
Concurrent TMZ + TTF + bevacizumab (*n* = 3)	1 vs 2	0 vs 3	1 vs 2	2 vs 1
Total (*n* = 60)	19 vs 41	7 vs 53	33 vs 27	31 vs 29

*IDH* isocytrate dehydrogenase, *MGMT* O-6-methylguanine-DNA methyltransferase, *TMZ* temozolomide, *TTF* tumor treating fields;

The 57 patients who started the AV-GBM-1 treatment received 392 injections, an average of 6.9 injections per patient. Thirty-nine patients received all eight injections (68.4%), three received seven doses, five received six doses, two received four, five received three, and three received two. All patients who discontinued treatment before the planned eight doses did so because of symptoms and MRI findings consistent with progressive disease, except one who stopped because of seizures and one who died from an unknown cause. Forty patients took corticosteroids concurrently with AV-GBM-1.

### Safety

Table 4 summarizes the most frequent treatment-emergent adverse events (TEAE). Only 26 AEs were attributed to AV-GBM-1, 24 grade-1, and 2 grade-2. (Supplementary Table 1). The most common AEs attributed to AV-GBM-1 were injection site reactions (15.8%), flu-like symptoms (10.5%), and bone pain (7%). An additional 74 AEs were classified as possibly due to AV-GBM-1, including many that are commonly observed in GBM patients treated with chemotherapy (Supplementary Table 2). One patient discontinued AV-GBM-1 because of seizures. There were 55 SAEs reported for 29 patients (Supplementary Table 3). These included hospitalizations for 16 episodes of seizures in 13 patients, seven falls in six patients, six episodes of focal weakness in four patients, and three patients with cerebral edema. One patient was discovered deceased at home after refusing to go to the hospital after a fall two days earlier, so the immediate cause of death was unclear.

**Table 4:** Most frequent treatment emergent adverse events (*n* = 57 patients)

Adverse Event	Grade 1 n (%)	Grade 2 n (%)	Grade 3 n (%)	Grade 4 n (%)	Total n (%)
Fatigue	20 (35.1)	10 (17.5)	1 (1.8)	0	31 (54.4)
Headache	13 (22.8)	6 (10.5)	2 (3.5)	0	21 (36.8)
Seizure	2 (3.5)	10 (17.5)	7 (12.2)	0	19 (33.0)
Nausea	11 (19.3)	6 (10.5)	0	0	17 (29.8)
Focal weakness	3 (5.3)	8 (14.0)	5 (8.7)	0	16 (28.1)
Thrombocytopenia	5 (8.7)	4 (7.0)	2 (3.6)	2 (3.6)	13 (22.8)
Insomnia	8 (14)	4 (7.0)	0	0	12 (21.0)
Vomiting	7 (12.2)	4 (7.0)	0	0	11 (19.3)
Abdominal pain	8 (14.0)	2 (3.5)	1 (1.8)	0	11 (19.3)
Fall	6 (10.5)	1 (1.8)	1 (1.8)	2 (3.5)	10 (17.5)
Dizziness	8 (14.0)	2 (3.5)	0	0	10 (17.5)
Cerebral edema	0	1 (1.8)	6 (10.5)	2 (3.5)	9 (15.8)
Injection-site reaction	8 (14.0)	1 (1.8)	0	0	9 (15.8)
Myalgia	5 (8.7)	3 (5.3)	0	0	8 (14.0)
Depression	4 (7.0)	4 (7.0)	0	0	8 (14.0
Neutropenia	0	2 (3.5)	4 (7.0)	1 (1.8)	7 (12.2)
DVT/PE	0	3 (5.3)	4 (7.0)	0	7 (12.2)
Flu-like symptoms	6 (10.5)	1 (1.8)	0	0	7 (12.2)
Confused/Forgetful	2 (3.5)	3 (5.3)	1 (1.8)	0	6 (10.5)
Bone pain	6 (10.5)	0	0	0	6 (10.5)
Pruritis	6 (10.5)	0	0	0	6 (10.5)

*DVT/PE* deep venous thrombosis and/or pulmonary embolus

### Efficacy

**Fig. 2:**
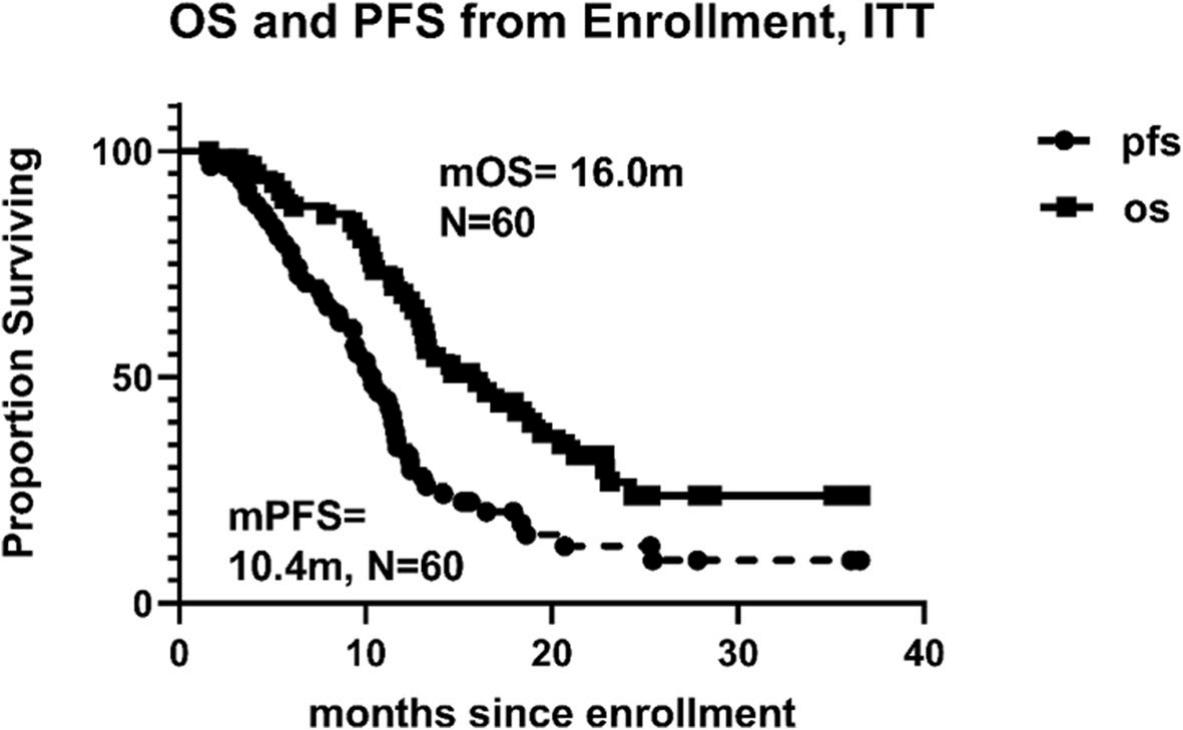
OS and PFS from intent-to-treat enrollment

Figure 2 shows OS and PFS survival curves, and Table 5 shows mOS and percentage OS data at 6-month intervals. From enrollment, OS at 14.6 months was 53%, consistent with the benchmark and well short of our primary aim goal of 75%. From enrollment, mPFS was 10.4 months (95%CI: 8.6 to 11.7), which is statistically greater than the benchmark figure of 6.9 months [5] (Table 5).

**Table 5:** Overall survival and progression-free survival dating from enrollment and, separately, first injection: median and six-month intervals (data cut off 31 Dec 21)

	From enrollment		From first injection	
	OS	PFS	OS	PFS
N (censored)	60 (21)	60 (10)	57 (19)	57 (9)
Median (months)	16.0	10.4	14.0	8.5
95% CI on Median	12.9:20.6	8.6:11.7	10.1:18.3	6.5:9.1
Percent Surviving* (remaining at risk at the indicated time)				
6 months	89.5%(51)	77.7%(45)	87.5%(49)	69.7%(39)
12 months	70.2%(40)	34.5%(20)	55.4%(31)	26.8%(15)
18 months	44.5%(20)	20.2%(9)	38.5%(16)	16.1%(6)
24 months	26.8%(9)	12.6%(5)	25.2%(7)	10.0%(3)

*If no date of progression, observation for progression ends on last date known alive

All *n* = 39 deceased are considered progressed

If no event at the indicated time, then percent surviving is as of the most recent event before the indicated time

### Exploratory analyses

OS was compared for a variety of subsets defined by the patient (age, KPS), tumor characteristics (MGMT promoter methylation and IDH1/2 mutation status), or anti-cancer therapy (number and cell composition of vaccinations received, TTF and/or bevacizumab use, and corticosteroid use). These comparisons were unadjusted, and the results must be regarded as exploratory.

In our study, higher KPS but not younger age did associate with better OS. mOS was 19.5 months for KPS of 90 or 100 (*n* = 29) as compared to 13.0 months for KPS of 70 or 80 (*n* = 31, *p* = 0.005). mOS was 19.5 months for 33 patients less than 60 years of age, compared to 13.0 months for 27 age 60 to 70 years (*p* = 0.123). Age at enrollment did not correlate with OS time (*r* = −0.12, *p* < 0.37).

mOS had not been reached for the seven patients whose tumors were *IDH*-mutated (3 deceased), compared to 14.7 months for 53 with *IDH* wild-type (*p* = 0.101). mOS was 16.5 months for 18 patients with a methylated *MGMT* promotor and IDH wild-type, compared to 14.6 months for 34 patients with unmethylated MGMT promoter and IDH wild-type (*p* = 0.096).

There was no correlation between OS and the number of ITC irradiated during vaccine production, the number of cryopreserved DC-ATA per dose, or post-cryopreservation number per dose. mOS was 20.5 months for 39 who completed all eight doses compared to 9.8 months for 18 who received 2 to 7 injections (*p* = 0.0001). Medians were 10.5 months for the eight who received six or seven injections and 5.5 months for the 10 who received 2 to 4 injections. This relation may reflect dose-response or simple reverse causality.

TTF has been shown to improve survival when administered with maintenance TMZ compared to TMZ alone [26]. The addition of TTF during maintenance TMZ (*n* = 7) was associated with a mOS of 14.7 months, compared to 18.1 months for 11 patients treated with TMZ plus bevacizumab, 20.5 months for 28 patients treated with TMZ alone, and 13.0 months for 3 patients treated with TTF, TMZ, and bevacizumab. These differences in mOS are not statistically significant (*p* < 028).

Concomitant medication with corticosteroids might inhibit vaccine effects. The median OS for the 40 taking corticosteroids concurrently with the vaccine was 15.4 months compared to 20.5 months for the 17 who did not (*p* = 0.375).

## Conclusion

This trial examined (1) the efficiency of producing patient-specific DC-ATA vaccine AV-GBM-1 (2) AEs associated with u*p* to 8 s.c. injections of AV-GBM-1 admixed with adjuvant GM-CSF, and (3) OS and PFS for patients treated with AV-GBM-1 as an adjunct to SOC of nGBM.

Sufficient tumor cells and monocytes were collected for 97% of patients, and the success rate for converting these into the final AV-GBM-1 product was 100%. The screening of 106 patients resulted in the collection of 80 GBM tumors and enrollment of 60 patients for a 56.6% rate based on screening and a 75% rate based on GBM tumor collection, which compares favorably to the 20.7% rate demonstrated in the recent publication of a large previous trial of the DCVax-L autologous tumor cell vaccines [28].

AV-GBM-1 was well-tolerated, but there were a high number of TEAEs mainly attributed to other concomitant therapeutic agents or complications of GBM. The 9/57 (15.8%) rate of mild to moderate local injection site reactions was much lower than the 55/72 (76%) rate (*p* < 0.0001) observed in patients with metastatic melanoma [10], which may be due to immunosuppressive effects of corticosteroids taken by the majority of GBM patients. Even though GBM patients are expected to have central nervous system AEs because of their cancer, the rate of seizures was higher than reported in other trials [29, 30]. Compared to the 19/57 (33.0%) proportion reporting seizures in association with AV-GBM-1, in these two trials, the proportions of patients with convulsions were much less, 28/258 (11%) (*p* = 0.0001) [29], and 69/372 (18%) (*p* = 0.0136, [30]. This data suggests that AV-GBM-1 may have induced an immune response that increased inflammation at the tumor site, potentially inducing seizures in some patients. This merits further research.

Encouraging results for autologous DC incubated with lysates from fresh whole tumors were reported from other small phase II trials [31, 32]. However, two randomized trials in GBM failed to show a clear increase in mOS [28, 33] AV-GBM-1 is different because it uses antigens derived from autologous TICs rather than fresh whole tumors. The admixing of the DC-ATA product with GM-CSF also differentiates our approach from that of others. Our antigen-loaded DC are only partially matured during incubation with ATA and are believed to complete maturation only after the s.c. injection with GM-CSF. In our study, the mOS was 16.0 months, a 10% increase in mOS compared to the survival benchmark (14.6 months) reported in the original Stupp trial [5]. Our 2 year OS (27%) is lower than the results of the recently published phase 3 DCVax-L study (35%). However, the DCVax-L study excluded a large number of patients with potential disease progression/pseudo-progression (PD/PsPD) after XRT + TMZ (250 patients excluded due to post-XRT MRI changes versus 331 patients randomized) while our study allowed all the patients to stay in the clinical trial. The GBM patients with PD immediately after XRT have a significantly worse prognosis than the patients with stable disease or tumor response and the presence of these patients in our study could explain the OS difference.

The modest mOS could be possibly also due to the fact the vaccine administration was limited to a maximum of eight doses over six months in this protocol, while previous studies allowed for vaccination over more extended periods of time.

AV-GBM-1 was associated with an increased mPFS, about 50% longer than historical benchmarks [3–6]. The mPFS from enrollment is 10.4 months pre-RT/TMZ (8.6, 11.7, 95% CI), and 8.5 months from the first injection (6.5, 9.1, 95% CI,Table 5). Some of our patients received additional treatments such as bevacizumab and NovoTTF in addition to the standard adjuvant TMZ. Both bevacizumab [3] and NovoTTF [26] improve mPFS in randomized GBM clinical trials. However, in our study, the patients receiving NovoTTF did not display superior mPFS (8.7 months vs. 9 months from the first injection, *p* < 0.77). Similarly, the bevacizumab treatment did not improve mPFS in our patient population (8.2 months vs. 9.1 months from the first injection, *p* < 0.14). Five published studies included a SOC study arm and enrolled patients before RT/TMZ [3–6, 33]. In these trials, the mPFS was 6.9 months (5.8, 8.2 95% CI) [5], 7.3 months (5.9, 7.9, 95%CI) [4], 6.2 months (95 CI not reported) [3], 7.5 months (7.1, 8.0 95% CI) [6], and 6.9 months (5.9, 9.4 95% CI) [33]. The lower bound for AV-GBM-1 mPFS pre-RT/TMZ excludes the upper bound in four of those trials. Two published studies included a SOC arm but enrolled patients after recovery from RT/TMZ [26, 30]. In these trials, the mPFS was 4.0 months (3.8, 4.4 95% CI) and 5.6 months (5.1, 7.1 95% CI). The lower bound for AV-GBM-1 mPFS post RT/TMZ also excludes the upper bound in these two trials. Our mPFS from vaccination results seem comparable to the mPFS of 6.7 months (6.1, 8.1 95% CI) reported for the TTF arm of the pivotal randomized trial, which led to the TTF approval as SOC therapy for nGBM [26].

Our findings suggest that AV-GBM-1 is well-tolerated and might increases mPFS, and this should be investigated through larger, randomized trial. Given the success rate of producing the vaccine and the observation that most progression and death occurred after AV-GBM-1 was discontinued, increasing the number of treatment cycles in our future randomized clinical trial may confer a more significant survival benefit. A Phase 3 trial for AV-GBM-1 has been approved by the FDA, and this will permit a more consistent and randomized comparison of AV-GBM-1 to the SOC without any potential confusion created by differential application of concurrent treatment.

## Supplementary Information

**Additional file 1: Supplementary Data 1.** AV-GBM-1 production feasibility. **Supplementary Data 2.** Protocol Inclusion and Exclusion Criteria. **Supplementary Data 3.** Explanation Behind TIC Isolation Methodology

## Data Availability

The de-identified datasets created and analyzed during the current study are available from the corresponding author on reasonable request.

## Abbreviations

GBM: Glioblastoma, WHO grade 4 Malignant Glioma
AE: adverse events
RT/TMZ: concurrent Radiation Therapy and Temozolomide
ITT: intent-to-treat
TIC: Tumor-Initiating Cells
KPS: Karnofsky Performance Score
GM-CSF: Granulocyte Macrophage Colony-Stimulating Factor
mPFS: Median Progression-free Survival
mOS: Median Overall Survival
SOC: Standard of care
nGBM: Newly-diagnosed Glioblastoma
DC: Dendritic Cells
ATA: Autologous Tumor Antigens
SAEs: Serious Adverse Events
PD: Progressive Disease
TEAE: Treatment-emergent Adverse Events
MGMT: O-6-methylguanine-DNA methyltransferase
IDH 1 / 2: Isocitrate Dehydrogenase
TTF: Tumor Treating Fields
TPP: Target Product Profile
