# Supplementary material for: Phase 2 study of AV-GBM-1 (a tumor-initiating cell targeted dendritic cell vaccine) in newly diagnosed Glioblastoma patients: safety and efficacy assessment
Source: J Exp Clin Cancer Res. 2022 Dec 14;41:344. doi: 10.1186/s13046-022-02552-6 (PMC9749349; doi:10.1186/s13046-022-02552-6)
Supplement: Supplementary file 1 — Additional file 1: Supplementary Data 1. AV-GBM-1 production feasibility. Supplementary Data 2. Protocol Inclusion and Exclusion Criteria. Supplementary Data 3. Explanation Behind TIC Isolation Methodology [file 13046_2022_2552_MOESM1_ESM.docx]

**Supplemental Data:**

**Supplementary Data 1**. *AV-GBM-1 production feasibility*

The manufacturing and delivery of AV-GBM-1 included (1) coordination of tumor collection at the time of craniotomy, (2) successful establishment of a short-term culture of self-renewing TICs, (3) collection of PBMC by leukapheresis, (4) differentiation of monocytes into DC, (5) incubation of DC and a lysate of the autologous irradiated self-renewing TICs, and (6) coordination of shipping individual doses to treatment sites followed by vaccine injection within five hours of thawing.

The ATA source was a lysate of irradiated autologous TICs from a short-term cell culture derived from the patient’s GBM. Fresh tumor tissue was collected at the time of surgery and transported by overnight courier to AIVITA Biomedical, Inc. (Irvine, CA). Each tumor sample was dissociated into a single-cell suspension, then incubated in proprietary serum-free media to produce tumor spheroids and eliminate lymphocytes and other hematopoietic cells, stromal cells and connective tissue. Cultures were continued for 28 days or to 100 million cells, whichever came first. Cells were then irradiated at 100 Gy in a Precision CellRad x-ray cell irradiator (Precision X-Ray, Branford, CT). After irradiation, the “stressed” tumor cells were placed back into culture for 18 to 24 hours to allow synthesis and expression of damage-associated molecules that attract DC for phagocytosis, then frozen and thawed repeatedly to create a tumor cell lysate that was cryopreserved in the vapor phase of liquid nitrogen.

After recovery from surgery, patients underwent leukapheresis to collect peripheral blood mononuclear cells (PBMC) that were shipped by overnight courier to the AIVITA manufacturing facility. The PBMC product was further enriched for monocytes using the Elutra® Cell Separation System (Terumo BCT, Lakewood, CO.). If fewer than 450 million cells were collected, an additional leukapheresis was performed if the patient was willing to do so. The monocytes (MC) were then incubated for six days in media containing the cytokines GM‑CSF (Leukine®, Partner Therapeutics) and interleukin-4 (CellGenix, Portsmouth, NH) to differentiate MC into DC. The final product was produced by incubating DC and ATA lysate together for 24 hours. The final product was tested for microbial contamination, *Mycoplasma*, and endotoxin.

46/60 (77%) tumors were in culture for 28 days or less; 11 were in culture for 30 to 35 days, and the remaining three were cultured 46, 54, and 55 days. 58/60 (97%) cultures yielded more than 1 million TICs for irradiation prior to creating the tumor cell lysate and more than 10 million cells were irradiated for 36 (60%). More than 450 million monocytes were cryopreserved for differentiation into DC for 52 patients (87%). There were more than 1 million cells per DC-ATA dose for 51 patients (85%), with viabilities greater than 50% for 59 (98%) and greater than 70% for 48 (80%). Of the eight patients who had fewer than 450,000 monocytes from the leukapheresis products, only three had fewer than 1 million viable DC-ATA per dose based on the final product.

**Supplementary Data 2** *Protocol Inclusion and Exclusion Criteria*

Intent-to-Treat Enrollment Phase Inclusion Criteria

1 Age ≥ 18 years of age.

2 KPS ≥ 70

3 Successful establishment of an autologous cancer cell line by AIVITA Biomedical, Inc.

4 Confirmation of GBM histology.

5 Completed a leukapheresis procedure from which PBMC were successfully collected.

6 Written informed consent for treatment with investigational treatment.

7 Patients may proceed with AV-GBM-1 treatment regardless of the interpretation of a post CT/RT MRI. It is recognized that some patients will have a scan that seems to show early progression, and that for some of the patients it is actually only inflammation (pseudoprogression) that will subsequently resolve while for other patients the early imaging changes are consistent with true progression.

8 Patient may be enrolled even if they are taking up to the equivalent of 4 mg dexamethasone, but ideally, they will be on tapering or physiologic doses rather than pharmacologic doses, at that time of treatment, which will be probably be about 10 to 13 weeks after enrollment, and about 4 to 6 weeks after completion of concurrent CT/RT.

Treatment Phase Exclusion Criteria

1 Known to have active hepatitis B or C or HIV.

2 KPS < 70

3 Known underlying cardiac disease associated with myocardial dysfunction that requires active medical treatment, or unstable angina related to atherosclerotic cardiovascular disease, or under treatment for arterial or venous peripheral vascular disease.

4 Diagnosis of any other invasive cancer or other disease process which is considered to be life-threatening within the next five years, and/or taking anti-cancer therapy specific for cancer other than GBM (such as continuation of hormonal therapy for breast cancer diagnosed more than five years earlier). The Chief Medical Officer of AIVITA Biomedical, Inc. may grant approval on a case by case basis for patients who have previously been treated for malignancy.

5 Active infection or other active medical condition that could be eminently life-threatening, including active blood clotting or bleeding diathesis.

6 Known autoimmune disease, immunodeficiency, or disease process that involves the use of immunosuppressive therapy.

7 Received another investigational drug within 28 days of the first dose or planning to receive another investigation drug while receiving this investigational treatment.

8 Known hypersensitivity to GM-CSF.

9 Pregnancy

**Supplementary Data 3** *Explanation Behind TIC Isolation Methodology*

It is well-known that the immunogenic properties of the TICs, or cancer cells in general, do not reside in common markers (i.e. nestin, Sox2, vimentin, CD44, Ki67, CD133 etc). These are normal proteins expressed by normal cells, having no immunogenic properties, however, their secretion is dysregulated and found consistently overexpressed in GBM. The immunogenic component of the tumor resides in neo-antigens resulting from a variety of mutations during tumorigenesis. These neoantigens do not have classic markers identified by histological staining methods. Currently, neoantigens are identified by DNA or RNA investigative methods. The obtained sequences are then compared to normal genomes or known mutation databases. The antigenic properties of hundreds and occasionally thousands of mutated peptides were investigated by theoretical matching with HLA databases, however the immunogenic effectiveness was not consistently confirmed by in vivo experiments or clinical trials. The advantage to use a purified population of TICs consist in a better signal (neoantigens) to noise (all other proteins), the use of transitory expressed neoantigens that cannot be identified in senescing tumor cells and the use of entire “mutanome” allowing autologous APC selection of the “best fit” antigen.

In current knowledge, tumor initiating cells (TIC) are considered broadly equivaled with the concept of “stem cells”, having self-renewal as a common biological property. Expanding on stem cell analogy, it is thought that tumor initiating cells should have at least some normal stem cell markers and, according to some scholars, should have the originating tissue’s stem cell multipotency. In cancer cell biology research, the “multipotent stem cell” theory failed in general, thus the equivalence of TIC with normal stem cells resumes exclusively to the self-renewal characteristic. One of the fundamental reasons is the nature of self-renewal in normal stem cells as a developmental characteristic (ontogenesis), while in cancer is the result of immune editing of cell population that has a mutation with survival advantage. In tumorigenesis, mutated cells with stronger antigenic properties are systematically eliminated, while the ones with weak or non-recognizable neo-antigens resulting from subsequent branching mutations are spared. Depending on the trunk (driver) mutation, each tumor can evolve with unique phenotype, having “self-renewal” as the only obvious common characteristic. Efforts to establish a particular phenotype that is specific to TICs systematically failed. On the other hand, tumors maintain in general some the characteristics of the originating tissue (i.e. glial phenotype) and may upregulate certain components of the normal self-renewal machinery that are in general regulatory transcription factors (SOX2), growth factor receptors (FGFR, EGFR), transitory somatic markers such as CD133, Nestin, Vimentin, and adhesion molecules NCAM, CD44. We investigated the frequency of a variety of markers and found to agree with the waste GBM published literature.

In our manufacturing, the selection of an autologous immunogenic population in AV-GBM-1 vaccine is based on an “in-vitro” method of isolation and transitory amplification of the most resilient and proliferative cells from the tumor sample, based on the self-renewal property of TICs, not on a phenotypical marker. The method uses a serum free media designed to activate the amino-acid dependent mTOR pathways as opposed to other published methods using growth factors from receptor tyrosine kinase family (i.e. EGF/FGF). As consequence of the “survival of the fit” approach, the in-vitro selected cells consist in a homogenous population with high mitotic index as shown by positivity to Ki67. Most cells (over 95%) are positive for Nestin and Vimentin and express homophilic adhesion molecules (NCAM, PS-NCAM) that confer the well-know “spherogenic” characteristic of neural progenitors. In some cases the isolated population can display a mesenchymal transition (EMT) expressing CD44. The self-renewal property of the AV-GBM-1 immunogenic TIC population was demonstrated by an established clonogenic assay, in which cells cultured at clonal densities can survive and expand. This demonstrates that the mechanism of self-renewal of TICs obtained by our methods is not driven by paracrine signals (i.e. wingless and/or hedgehog) or growth factor mediated, but rather by a transcriptional dysregulation.

Supplemental Table 1

Adverse events which investigators attributed to AV-GBM-1 (DC-ATA/GM-CSF)

| Adverse Event | Grade 1  n (%) | Grade 2  n (%) | Grade 3  n (%) | Grade 4  n (%) | Grade 5  n (%) | Total  n (%) |
| --- | --- | --- | --- | --- | --- | --- |
| Injection-site reaction | 8 (14.0) | 1 (1.8) | 0 | 0 | 0 | 9 (15.8) |
| Flu-like symptoms | 6 (10.5) | 0 | 0 | 0 | 0 | 6 (10.5) |
| Bone pain | 4 (7.0) | 0 | 0 | 0 | 0 | 4 (7.0) |
| Left arm cellulitis | 0 | 1 (1.8) | 0 | 0 | 0 | 1 (1.8) |
| Arthralgia (joint pain) | 1 (1.8) | 0 | 0 | 0 | 0 | 1 (1.8) |
| Myalgia (muscle pain) | 1 (1.8) | 0 | 0 | 0 | 0 | 1 (1.8) |
| Pruritis | 1 (1.8) | 0 | 0 | 0 | 0 | 1 (1.8) |
| Bruising | 1 (1.8) | 0 | 0 | 0 | 0 | 1 (1.8) |
| Memory loss | 1 (1.8) | 0 | 0 | 0 | 0 | 1 (1.8) |
| Lower quadrant pain | 1 (1.8) | 0 | 0 | 0 | 0 | 1 (1.8) |

DC-ATA=vaccine of autologous dendritic cells pulsed with autologous tumor antigens from lysate of irradiated self-renewing tumor cells

GM-CSF=granulocyte macrophage colony stimulating factor

Supplemental Table 2

Adverse events which investigators felt were possibly related to AV-GBM-1

| Adverse Event | Grade 1  n (%) | Grade 2  n (%) | Grade 3  n (%) | Grade 4  n (%) | Grade 5  n (%) | Total  n (%) |
| --- | --- | --- | --- | --- | --- | --- |
| Fatigue | 11 (19.3) | 4 (7.0) | 0 | 0 | 0 | 15 (26.3) |
| Seizure | 2 (3.5) | 5 (8.8) | 3 (5.2) | 0 | 0 | 10 (17.4) |
| Nausea | 5 (8.7) | 2 (3.5) | 0 | 0 | 0 | 7 (12.2) |
| Cerebral edema | 0 | 1 (1.8) | 3 (5.2) | 1 (1.8) | 0 | 5 (8.8) |
| Headache | 4 (7.0) | 0 | 1 (1.8) | 0 | 0 | 5 (8.8) |
| Vomiting | 5 (8.8) | 0 | 0 | 0 | 0 | 5 (8.8) |
| Myalgia (muscle pain) | 4 (7.0) | 0 | 0 | 0 | 0 | 4 (7.0) |
| Platelet count decreased | 1 (1.8) | 1 (1.8) | 0 | 1 (1.8) | 0 | 3 (5.2) |
| Weakness/Rt or Lt side | 0 | 1 (1.8) | 2 (3.5) | 0 | 0 | 3 (5.2) |
| Rash | 0 | 1 (1.8) | 1 (1.8) | 0 | 0 | 2 (3.5) |
| Fall | 1 (1.8) |  | 1 (1.8) | 0 | 0 | 2 (3.5) |
| Arthralgia (joint pain) | 1 (1.8) | 1 (1.8) | 0 | 0 | 0 | 2 (3.5) |
| Confusion/forgetfulness | 0 | 2 (3.5) | 0 | 0 | 0 | 2 (3.5) |
| Non cardiac chest pain | 1 (1.8) | 1 (1.8) | 0 | 0 | 0 | 2 (3.5) |
| Bone pain | 2 (3.5) | 0 | 0 | 0 | 0 | 2 (3.5) |
| Pruritis | 2 (3.5) | 0 | 0 | 0 | 0 | 2 (3.5) |
| Abdominal pain/cramps | 2 (3.5) | 0 | 0 | 0 | 0 | 2 (3.5) |
| GERD | 2 (3.5) | 0 | 0 | 0 | 0 | 2 (3.5) |
| Hyponatremia | 2 (3.5) | 0 | 0 | 0 | 0 | 2 (3.5) |
| Parasthesia | 2 (3.5) | 0 | 0 | 0 | 0 | 2 (3.5) |
| Pancreatitis | 0 | 0 | 1 (1.8) | 0 | 0 | 1 (1.8) |
| Memory loss | 0 | 0 | 1 (1.8) | 0 | 0 | 1 (1.8) |
| PE/DVT | 0 | 0 | 1 (1.8) | 0 | 0 | 1 (1.8) |
| Nose bleeds | 0 | 0 | 1 (1.8) | 0 | 0 | 1 (1.8) |
| Flu-like symptoms | 0 | 1 (1.8) | 0 | 0 | 0 | 1 (1.8) |
| Double/Blurred vision | 0 | 1 (1.8) | 0 | 0 | 0 | 1 (1.8) |
| Pyramidal tract syndrome | 0 | 1 (1.8) | 0 | 0 | 0 | 1 (1.8) |
| Constipation | 0 | 1 (1.8) | 0 | 0 | 0 | 1 (1.8) |
| Hypothyroidism | 0 | 1 (1.8) | 0 | 0 | 0 | 1 (1.8) |
| Hypophosphatemia | 0 | 1 (1.8) | 0 | 0 | 0 | 1 (1.8) |
| Adrenal insufficiency | 0 | 1 (1.8) | 0 | 0 | 0 | 1 (1.8) |
| Dysarthria | 0 | 1 (1.8) | 0 | 0 | 0 | 1 (1.8) |
| Flatulence | 0 | 1 (1.8) | 0 | 0 | 0 | 1 (1.8) |
| Dyspnea (short of breath) | 0 | 1 (1.8) | 0 | 0 | 0 | 1 (1.8) |
| Lymphocytes decreased | 1 (1.8) | 0 | 0 | 0 | 0 | 1 (1.8) |
| Elevated creatinine | 1 (1.8) | 0 | 0 | 0 | 0 | 1 (1.8) |
| Fever | 1 (1.8) | 0 | 0 | 0 | 0 | 1 (1.8) |
| Tachycardia | 1 (1.8) | 0 | 0 | 0 | 0 | 1 (1.8) |
| Left hand edema | 1 (1.8) | 0 | 0 | 0 | 0 | 1 (1.8) |
| Depression | 1 (1.8) | 0 | 0 | 0 | 0 | 1 (1.8) |
| Extrapyramidal disorder | 1 (1.8) | 0 | 0 | 0 | 0 | 1 (1.8) |
| Speech aphasia | 1 (1.8) | 0 | 0 | 0 | 0 | 1 (1.8) |
| Facial droop | 1 (1.8) | 0 | 0 | 0 | 0 | 1 (1.8) |
| Insomnia | 1 (1.8) | 0 | 0 | 0 | 0 | 1 (1.8) |
| Eye irritation | 1 (1.8) | 0 | 0 | 0 | 0 | 1 (1.8) |
| Earwax buildup | 1 (1.8) | 0 | 0 | 0 | 0 | 1 (1.8) |
| Oral thrust | 1 (1.8) | 0 | 0 | 0 | 0 | 1 (1.8) |
| Hot flashes | 1 (1.8) | 0 | 0 | 0 | 0 | 1 (1.8) |

Supplemental Table 3.

Summary of Severe Adverse Events

**55 SAE among 29 patients; no SAE attributed to study agent**

16 hospitalized for seizures (3 patients twice)

7 hospitalized after falls, usually with other neuro complaints or findings (1 patient twice)

6 hospitalized for increased focal weakness (1 three times)

3 hospitalized for pancreatitis—(all 3 same patient)

3 hospitalized for other symptoms attributed to cerebral edema/mass effect (headache, visual)

3 hospitalized for pulmonary embolus & deep venous thrombosis

3 hospitalized for RUQ & fever, cholecystitis, sepsis (all 3 same patient)

2 hospitalized for altered mental status due to increased cerebral edema related to Ommaya (same patient twice)

2 hospitalized for chest pain and SOB

2 hospitalized for general deterioration

1 hospitalized for chest pain and fever

1 hospitalized for suicidal ideation

1 hospitalized for hyponatremia

1 hospitalized for increasing weakness that was secondary to pancytopenia

1 hospitalized for fever and weakness

1 hospitalized for facial edema

1 hospitalized for appendicitis

1 found dead at home after a fall two days earlier; had refused to go to hospital

Apheresis and monocyte intermediate products.

|  | Mean | Median | Lower range | Upper range |
| --- | --- | --- | --- | --- |
| Total Leukocytes x 10^9^ | 13.2 | 13.1 | 1.4 | 28.8 |
| Total Monocytes x 10^9^ | 2.0 | 1.8 | 0.15 | 5.4 |
| % CD14+/CD45+ | 66.4% | 67.5% | 23.3% | 95.2% |
| % Granulocytes | 21.8% | 17.0% | 1.9% | 61.8% |
| % Monocytes viable at freezing | 96.0% | 97.0% | 86% | 100% |
| # Viable monocytes frozen x 10^9^ | 1.7 | 1.5 | 0.75 | 5.2 |
